# The prognostic value of hedgehog signaling in bladder cancer by integrated bioinformatics

**DOI:** 10.1038/s41598-023-33140-z

**Published:** 2023-04-17

**Authors:** Xin Yu, Wenge Li, Yanjun Feng, Zhijie Gao, Qi Wu, Yue Xia

**Affiliations:** 1 Department of Breast and Thyroid Surgery, Renmin Hospital of Wuhan University, Wuhan, Hubei People’s Republic of China; 2 Department of Oncology, Shanghai Artemed Hospital, Shanghai, People’s Republic of China; 3 Tongji University Cancer Center, Shanghai Tenth People’s Hospital of Tongji University, School of Medicine, Tongji University, Shanghai, People’s Republic of China; 4 Department of Urology, Renmin Hospital of Wuhan University, Wuhan, Hubei People’s Republic of China

**Keywords:** Bladder cancer, Tumour biomarkers, Tumour immunology

## Abstract

Bladder cancer is the second most prevalent urological malignancy. It's a big contributor to cancer-related deaths throughout the globe. Researchers discovered that the hedgehog signaling (HhS) pathway contributed to the onset and spread of many different kinds of cancer. Nevertheless, the present understanding of the function of HhS in the bladder cancer molecular landscape is incomplete. Raw data were gotten from the IMvigor210, the Gene Expression Omnibus, and The Cancer Genome Atlas databases. Bioinformatics was used to examine the HhS score of each sample, and the enrichment of differentially expressed genes (DEGs), differentiation characteristics, immunological infiltration, and metabolic activity. The HhS prognostic signature was developed with significant assistance from the least absolute shrinkage and selection operator regression and Cox regression. An HhS-related nomogram was developed to assist in the prediction of patients’ survival probability. We found that HhS was linked to poor prognosis in bladder cancer, and its activation was linked to the Basal subtype of bladder cancer. Bladder cancer with high HhS activity has higher glycolysis, nucleotide metabolism, amino acid metabolism, and other cancer-promoting metabolic activities. Furthermore, HhS mediates an immunosuppressive microenvironment in bladder cancer on the basis that HhS negatively correlates with the CD8 + T cells and correlates positively with immune checkpoints and T cell exhaustion scores. Finally, an HhS-related signature was developed for predicting the prognosis of patients with bladder cancer. Targeting HhS may be a potential therapy choice for bladder cancer.

## Introduction

Bladder cancer is the second most prevalent urological cancer. It is also one of the leading causes of cancer-related deaths worldwide today. According to statistics, there are more than 500,000 new cases of bladder cancer and approximately 213,000 bladder cancer-related deaths each year^1^. Although a variety of treatments have been developed, including neoadjuvant, adjuvant chemotherapy, immunotherapy, and targeted therapies, resulting in a high survival rate for bladder cancer. However, due to the propensity for recurrence and metastasis, most patients must undergo lifelong surveillance^2,3^. Despite having similar clinical and histopathological features, bladder cancer exhibits substantial variation in disease aggressiveness and response to therapy due to tumor heterogeneity^2,3^. Therefore, investigations of the relevant biomarkers with predictive value for clinical outcomes and treatment efficacy to develop reliable tools to guide individualized treatment need to be expedited.

Hedgehog (Hh) was originally discovered in the early 1980s^4^ in Drosophila by Nüsslein-Volhard and Wieschaus. Binding among Indian hedgehog (IHH), Sonic hedgehog (SHH), and Desert hedgehog (DHH) ligands attenuates the suppressive effect of their Patched (PTCH) transmembrane proteins/receptors on Smoothened (SMO), which is also located in the cell membrane^5^. The signaling cascade initiated by SMO culminates in the activation and nuclear deployment of GLI gene transcription elements, thus inducing the expression of Hh target genes; the majority of these genes are involved in proliferation, survival, and angiogenesis. The Hh signaling (HhS) pathway is aberrantly activated by overexpression of Hh ligands, loss of receptor function, or dysregulation of transcription factors^5^. Evidence suggests that The constitutive activation of the HhS pathway is definitely linked to the onset and progression of several types of cancer, such as lung, gastric, prostate, hepatocellular, pancreatic, colorectal, esophageal, and ovarian cancers^6,7^. Moreover, evidence shows that multiple uroepithelial cancer cell lines exhibit constitutive HhS^8^. Given the functional significance of HhS in cancer and its crucial role in the disease, novel strategies targeting this pathway seem to hold promising therapeutic options for bladder cancer treatment^9^. However, the current comprehension of the function of HhS in the bladder cancer molecular landscape is incomplete.

The aim of this study is to determine HhS's predictive value for clinical outcomes in bladder cancer, analyze its phenotypic characteristics, and its potential impact on the immune environment and molecular landscape of bladder cancer. Finally, to construct and validate an HhS prognostic signature, thereby offering novel perspectives for the development of personalized therapy for bladder cancer.

## Materials and methods

### Data processing

**Figure 1:**
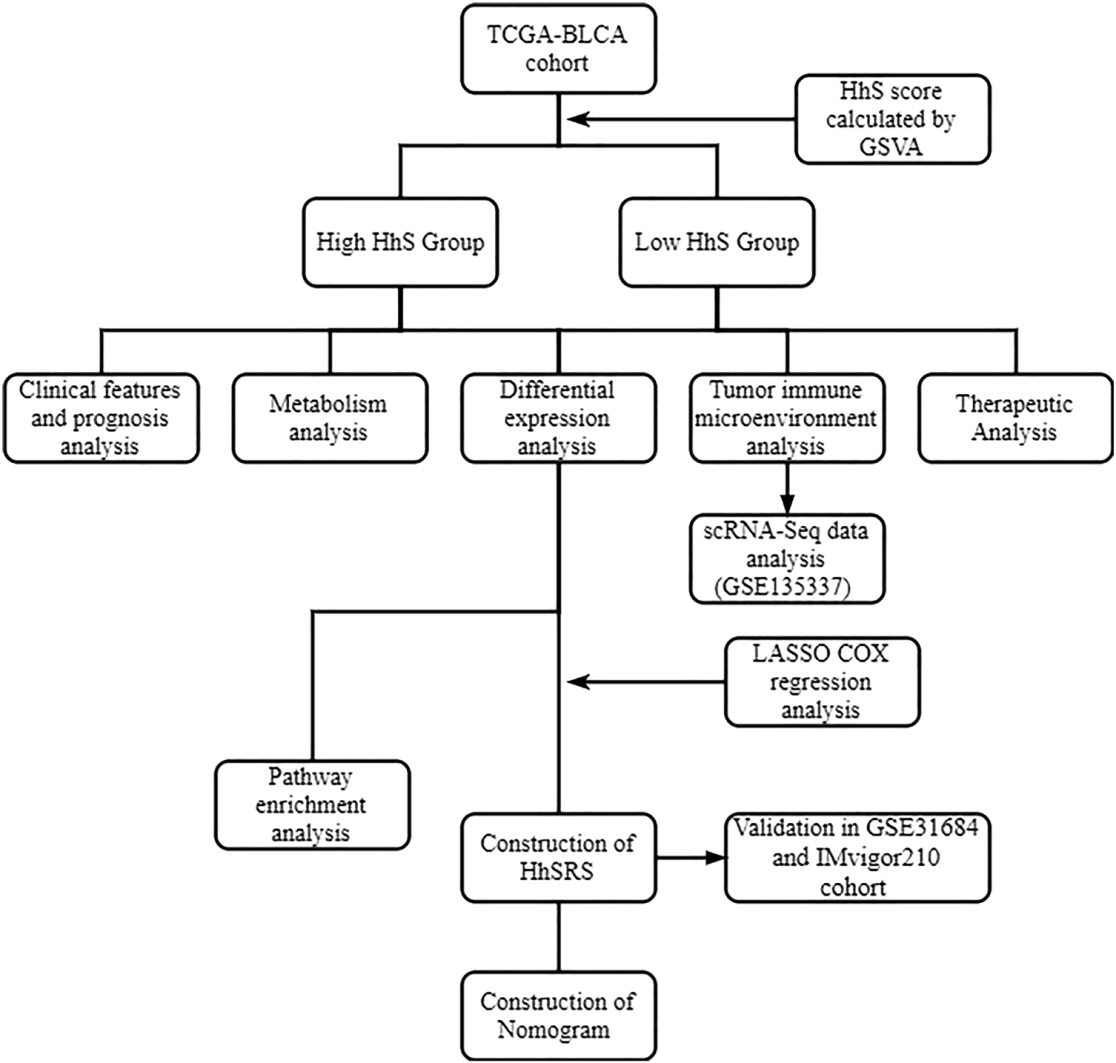
The framework of study design.

Figure 1 clearly shows the study’s framework design. For TCGA-BLCA, RNA-seq data from 408 BLCA patients in fragments per kilobase of exon per million mapped fragments (FPKM) values and matched medical data were retrieved from the UCSC Xena’s data portal. Subsequently, FPKM values were converted into transcripts per kilobase million (TPM) values. Somatic mutation data were obtained utilizing the R package TCGAbiolinks^10^ and then grouped in the Mutation Annotation Format (maf) form, followed by evaluation using the R package maftools.

From GEO, GSE31684 datasets ^11^ contained 93 bladder cancer samples that were used for external validation. In addition, a single-cell RNA sequencing (scRNA-seq) dataset of bladder cancer (GSE135337^12^) was used for scRNA-seq analysis.

For the BLCA immunotherapy-related cohort, the complete expression data as well as the detailed clinical information of 348 BLCA patients that received atezolizumab treatment were obtained by using the R package IMvigor210CoreBiologies^13^.

### Computation of the enrichment scores of different gene signatures

Utilizing nonparametric and unsupervised methods, gene set variation analysis (GSVA) can predict the specific pathway’s activity based on transcriptomic data^14^. HhS gene signature were acquired from the Molecular Signatures Database (MsigDB, https://www.gsea-msigdb.org/gsea/downloads.jsp) hallmark gene set collection The Bladder Cancer Molecular Taxonomy Group-related study yielded 12 bladder cancer signatures that are specific to various molecular subtypes ^15^. Metabolism-related gene sets were obtained from KEGG gene set collections on MsigDB. Therapeutic-related signatures, including immune-inhibited oncogenic pathways, targeted therapy-linked gene signatures, and gene signatures anticipating radiotherapy responses were collected from the study of Hu et al.^16^. All the gene sets are shown in Table S1.

### Screening and functional annotation of differentially expressed genes (DEGs)

DEGs were screened by the limma R package. *P* < 0.05 and fold change (FC) > 3/2 were set as the selection conditions for screening the downregulated and upregulated DEGs. The ClusterProfiler package performed Gene Ontology (GO), KEGG enrichment, and hallmark gene set enrichment. The R package ClusterProfiler, on the other hand, was used to perform gene set enrichment analysis (GSEA).

### Prediction of the molecular subtypes in bladder cancer

ConsensusMIBC R packages was utilized to ascertain the molecular subtypes of bladder cancer in different molecular subtype systems, encompassing CIT, MDA, Lund, Baylor, TCGA, and UNC subtypes.

### Evaluating immune cell infiltration

CIBERSORT, which can identify 22 tumor-infiltrating immune cells from each specimen, was used to measure the cellular density of the immune cells. The cancer immunity cycle gene sets can be grouped into seven stages: Stage 1 is the production of cancer cell antigens, Stage 2 is the presentation of cancer antigen, Stage 3 is the priming and activation, Stage 4 is the trafficking of immune cells to tumors, Stage 5 is the infiltration of immune cells to tumors, Stage 6 is the identification of cancer cells by T cells, and Stage 7 is the killing of cancer cells^17^. Then GSVA calculated the score of these gene sets.

### Predicting response to chemotherapy

The IC50 of common chemotherapeutic drugs was estimated by using the “pRRophetic” R package. The Drugbank database (https://go.drugbank.com/) was employed in the screening of the drug-target genes.

### Construction of the HhS-related signature (HhSRS)

The HhSRS was developed on the basis of the TCGA-BLCA cohort. The analysis of the univariate Cox regression was performed to find genes linked to overall survival (OS) from DEGs between the low-and-high- HhS group. Next, the LASSO regression played a crucial function in selecting candidate genes. Then, utilizing the multivariate Cox regression, the regression coefficient as well as the multiple regression model of genes linked to survival were determined. In these steps, the HhSRS was developed as follows: Risk score = Σn1 coefi*xi. The HhSRS was used to divide the groups into low- and high-risk categories, with the median score serving as the cutoff value.

### Construction of the HhSRS-related nomogram

This study used a multivariate Cox analysis of HhSRS grouping and clinical variables to identify potential independent prognostic indicators. The HHSRS-related nomogram was then constructed using the regplot software, using age, stage, and HhSRS group as parameters. To further aid in the display of the closeness between the actual and expected OS, calibration curves were generated.

### scRNA-Seq data analysis

The Seurat R package was used to perform unsupervised clustering of single cells using the read count matrix as input. The cell clustering, quality control, and annotation of scRNA-seq data were performed as per the previous description^18^. CellChat was employed in the intercellular communication network-related analysis from scRNA-seq data. The cytotoxic signature and exhaustion score of T cells were from the study of Zheng et al.(Table S2)^19^.

### Statistical analysis

The Pearson correlation analysis played a crucial role in the examination of correlations between variables. A t-test was employed in the comparison of continuous variables among binary groups that conform to a normal distribution. For comparisons of more than two groups, Kruskal–Wallis tests were used to compare the differences. The log-rank test was used to uncover statistically significant differences, and the Kaplan–Meier approach generated survival curves for the subgroups in each data set. SPSS 22.0, SangerBox ( http://sangerbox.com )^20^ and R 4.0.0 implemented all statistical analyses. P values were two-sided. P values < 0.05 were statistically significant.

## Result

### HhS activation is a marker of poor prognosis in BLCA patients

**Figure 2:**
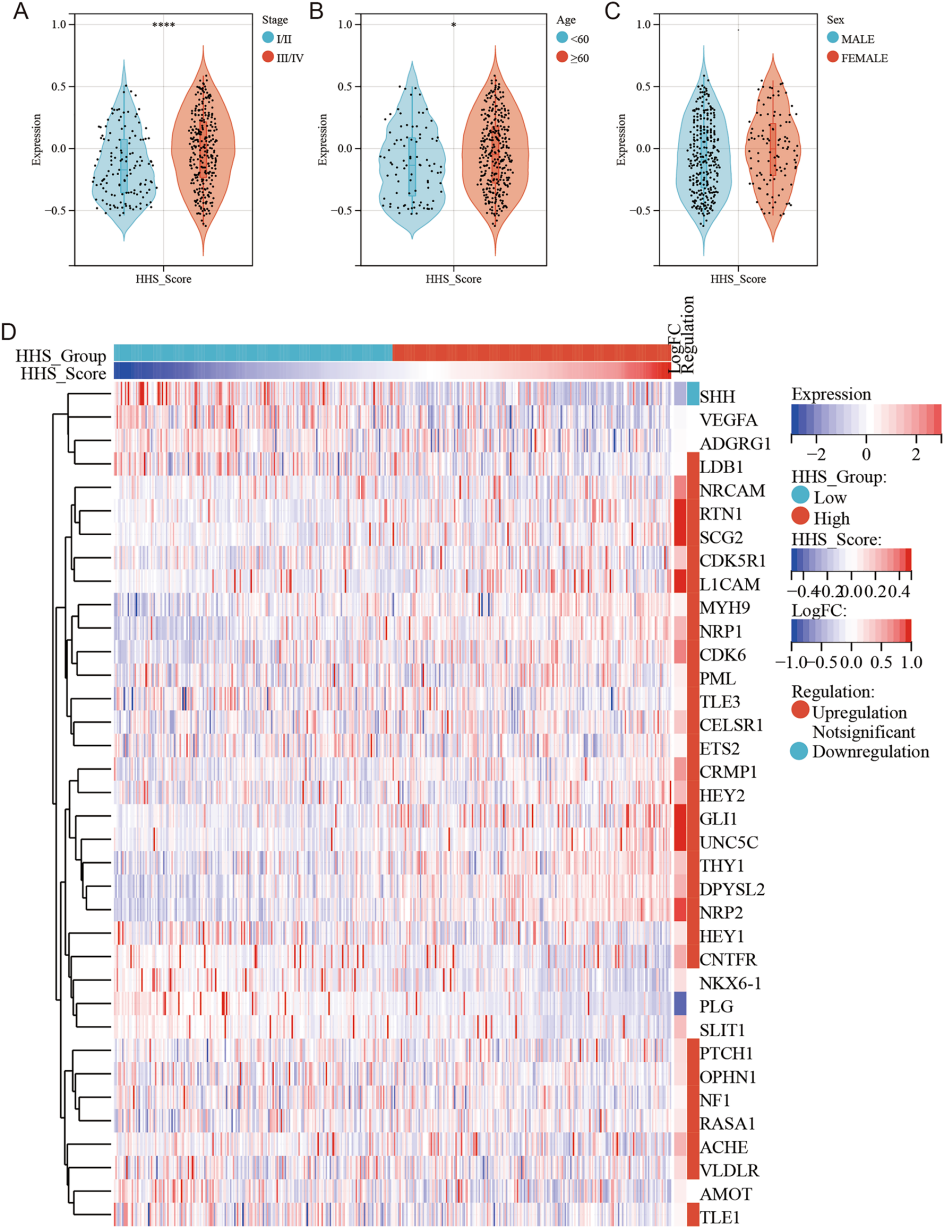
Expression of HhS in bladder cancer. (**A**–**C**) HhS score in different (**A**) stage, (**B**)Age and (**C**) sex. (**D**) Heatmap of HhS gene expression in high and low HhS score group patients. *P < 0.05, **P < 0.01, ***P < 0.001 and ****P < 0.0001.

First, HhS scores were calculated and its expression characteristics were analyzed for patients in the TGCA-BLCA cohort. The results showed that HhS was overactivated in patients with higher stage and older age (Fig. 2A, B). And there is no significant difference in HhS scores among patients of different genders (Fig. 2C). Next, patients were classified into a high-HhS group and a low-HhS group as per the median as the cut-off value (Fig. 2D). Variations in the expression of 36 genes involved in the HhS pathway were analyzed between the two groups of patients, and the findings affirmed that 28 of the 36 genes involved in HhS were remarkably expressed in the high HhS group (Fig. 2D).

**Figure 3:**
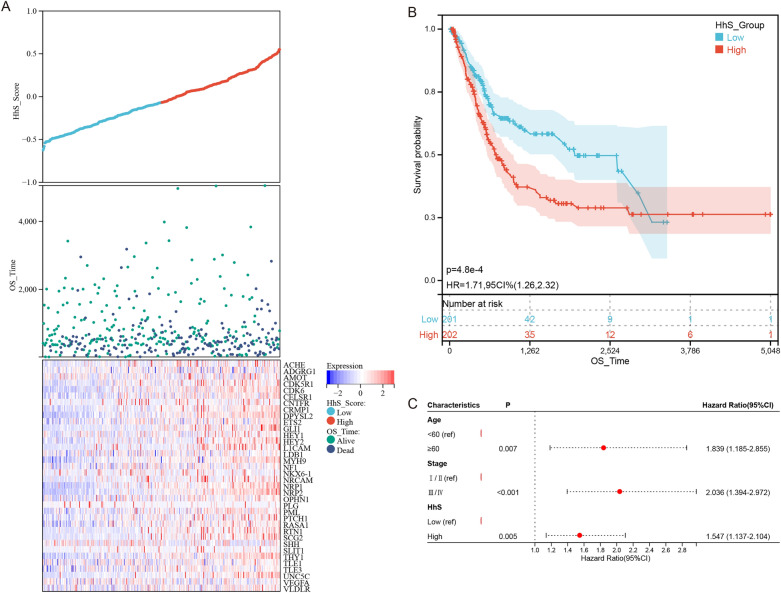
Prognosis value of HhS score in bladder cancer. (**A**) Risk score analysis, (**B**) Kaplan–Meier analysis and (**C**) Cox regression analysis for HhS score in OS of bladder cancer.

HhS scores, survival status, and expression of 36 HhS pathway genes in TCGA-BLCA patients were shown in Fig. 3A. KM analysis was performed and exhibited that the prognosis was worse in the high HhS group (Fig. 3B). Then, multivariate Cox regression analysis in the study, deduced that HhS acted as an independent prognostic f in bladder cancer patients (Fig. 3C).

These results indicated that HhS activation is a marker of poor prognosis in BLCA patients.

### Discovery of DEGs and functional annotations

**Figure 4:**
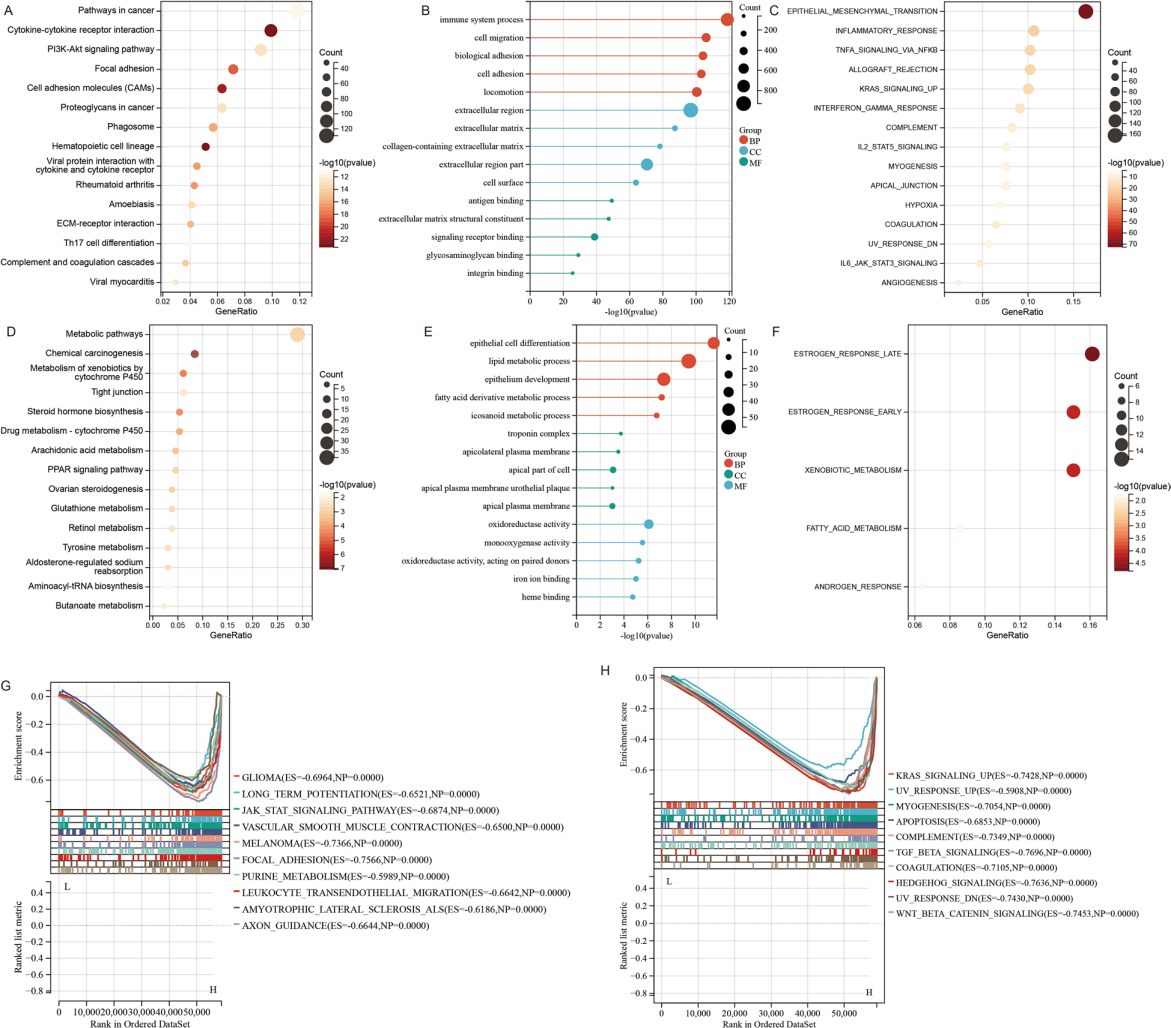
Functional annotation by KEGG, GO and Hallmark gene sets enrichment. (**A**–**C**) (**A**) KEGG, (**B**) GO and (**C**) Hallmark pathways of upregulated DEGs. (**D**–**F**) (**D**) KEGG, (**E**) GO and (**F**) Hallmark pathways of downregulated DEGs. (**G**–**H**) Functional analysis of HhS by GSEA based on gene sets from (**G**) KEGG and (**H**) Hallmark gene sets.

Regarding the DEGs, 551 downregulated DEGs and 2498 upregulated DEGs that belonged to the high HhS group were screened (Fig. S1A, B). Afterward, functional annotations of DEGs were performed. KEGG analysis showed that upregulated DEGs were mainly involved in “Pathways in cancer”, “Cytokine-cytokine receptor interaction” and “PI3K-Akt signaling pathway” (Fig. 4A). GO analysis demonstrated that upregulated DEGs were remarkably enriched in “Extracellular region” (cellular component, CC) “Immune system process” (biological process, BP), and “Antigen binding” (molecular function, MF) (Fig. 4B). The results of Hallmark gene set analysis showed that “Epithelial-mesenchymal transition”, “Inflammatory response”, and “TNFa signaling via NK-KB” (Fig. 4C).

For the downregulated DEGs, the KEGG pathways were “Chemical carcinogenesis”, “Metabolic pathways”, “Xenobiotics metabolism by cytochrome P450”, etc. (Fig. 4D). GO analysis manifested that the most substantially enriched pathways were “Epithelial cell differentiation” in BP, “Troponin complex” in CC, and “Oxidoreductase activity” in MF (Fig. 4E). And Hallmark gene sets analysis demonstrated that “Estrogen response early” “Estrogen response late”, and “Xenobiotic metabolism” were enriched (Fig. 4F).

Next, GSEA was performed and the results showed that the KEGG “Glioma”, “Long term potentiation”, and “JAK-STAT signaling pathway”, and the Hallmark “KRAS signaling up”, “UV response up” and “Myogenesis” were the most enriched in high HhS patients (Fig. 4G, H).

These results indicated that HhS was associated with differentiation characteristics, metabolism, and immunity in bladder cancer.

### Association between HhS and molecular characteristics

**Figure 5:**
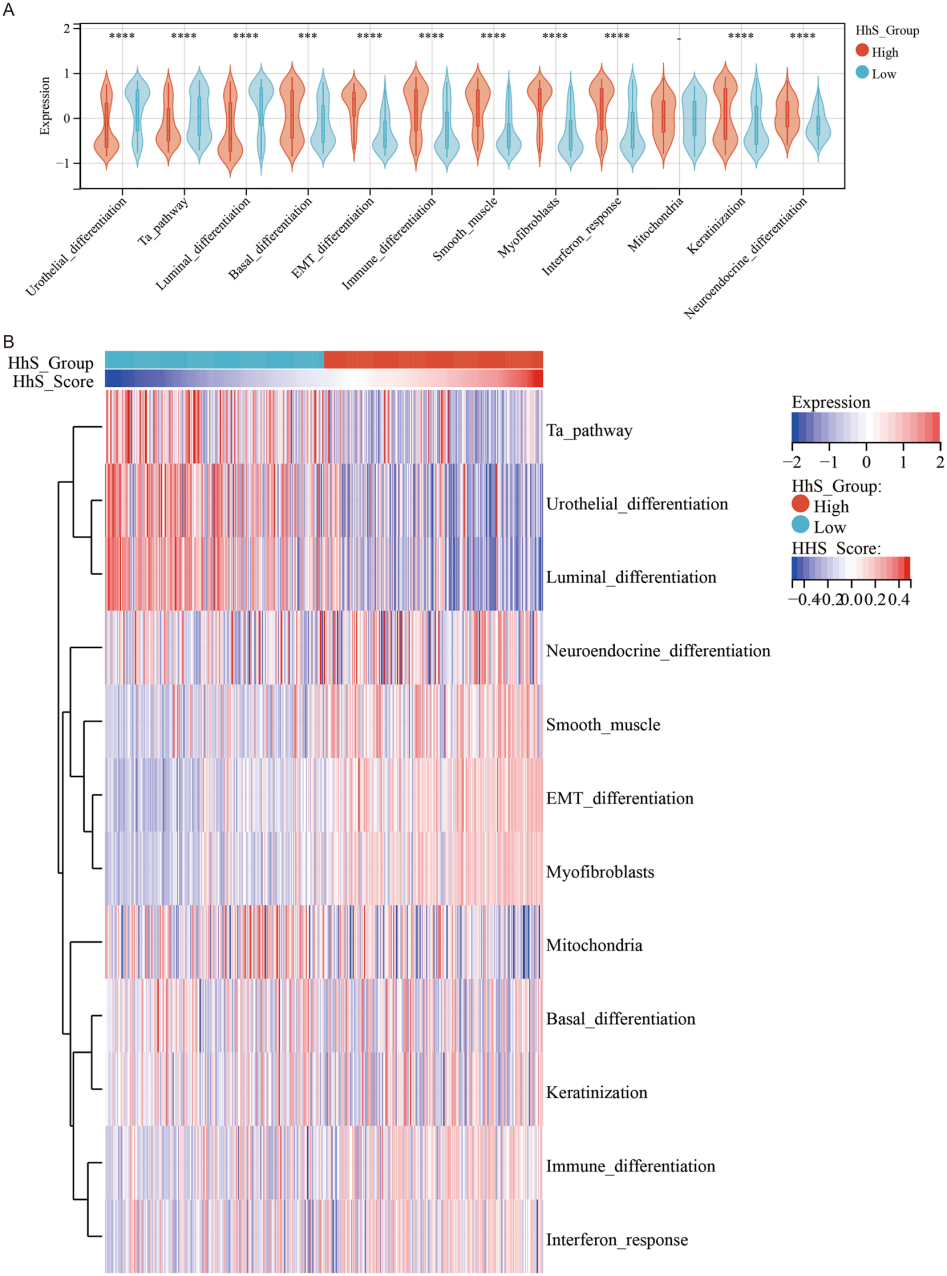
Correlation analysis between HhS and molecular subtype in bladder cancer. (**A**) Differential expression of specific bladder cancer-related signatures in high and low HhS score group patients. (**B**) Heatmap of specific bladder cancer-related signatures in high and low HhS score group patients. *P < 0.05, **P < 0.01, ***P < 0.001 and ****P < 0.0001.

The molecular subtypes of bladder cancer are closely linked to prognosis as well as response to chemotherapy, immunotherapy, and other therapies^15,21^. The association between HhS and molecular subtypes of bladder cancer was analyzed (Table. 1), and the results showed that six different molecular subtype prediction algorithms all suggest that bladder cancer with high expression of HhS is more likely to be basal type bladder cancer. And the analysis of molecular characteristics affirmed that the patients in the high HhS group had higher immune differentiation, basal differentiation, EMT differentiation, myofibroblasts, keratinization, smooth muscle, neuroendocrine differentiation, and interaction response scores, but lower urinary differentiation, Ta path and Luminal differentiation (Fig. 5A, B).

**Table. 1:** Correlations between HhS and molecular subtypes using six different algorithms and bladder cancer signatures.

Characteristics	Low(N = 204)	High(N = 204)	Total(N = 408)	*P* value
Baylor.subtype				**1.8e-9**
Basal	42(10.29%)	101(24.75%)	143(35.05%)	
Differentiated	162(39.71%)	103(25.25%)	265(64.95%)	
UNC.subtype				**1.2e-19**
Basal	44(10.78%)	136(33.33%)	180(44.12%)	
Luminal	160(39.22%)	68(16.67%)	228(55.88%)	
CIT.subtype				**1.3e-22**
MC1	127(31.13%)	35(8.58%)	162(39.71%)	
MC2	10(2.45%)	15(3.68%)	25(6.13%)	
MC3	14(3.43%)	4(0.98%)	18(4.41%)	
MC4	2(0.49%)	37(9.07%)	39(9.56%)	
MC5	0(0.0e + 0%)	1(0.25%)	1(0.25%)	
MC6	0(0.0e + 0%)	4(0.98%)	4(0.98%)	
MC7	51(12.50%)	108(26.47%)	159(38.97%)	
Lund.subtype				**1.1e-17**
Ba/Sq	14(3.43%)	39(9.56%)	53(12.99%)	
Ba/Sq-Inf	9(2.21%)	35(8.58%)	44(10.78%)	
GU	29(7.11%)	3(0.74%)	32(7.84%)	
GU-Inf	10(2.45%)	19(4.66%)	29(7.11%)	
Mes-like	5(1.23%)	29(7.11%)	34(8.33%)	
Sc/NE-like	6(1.47%)	11(2.70%)	17(4.17%)	
Uro-Inf	9(2.21%)	14(3.43%)	23(5.64%)	
UroA-Prog	71(17.40%)	23(5.64%)	94(23.04%)	
UroB	13(3.19%)	11(2.70%)	24(5.88%)	
UroC	38(9.31%)	20(4.90%)	58(14.22%)	
MDA.subtype				**3.9e-19**
Basal	40(9.80%)	103(25.25%)	143(35.05%)	
Luminal	116(28.43%)	29(7.11%)	145(35.54%)	
p53-like	48(11.76%)	72(17.65%)	120(29.41%)	
TCGA.subtype				**8.1e-25**
Basal_squamous	35(8.58%)	98(24.02%)	133(32.60%)	
Luminal	30(7.35%)	16(3.92%)	46(11.27%)	
Luminal_infiltrated	16(3.92%)	51(12.50%)	67(16.42%)	
Luminal_papillary	120(29.41%)	26(6.37%)	146(35.78%)	
Neuronal	3(0.74%)	13(3.19%)	16(3.92%)	

Significant values are in [bold ].

### Correlations between HhS and metabolism pathways

**Figure 6:**
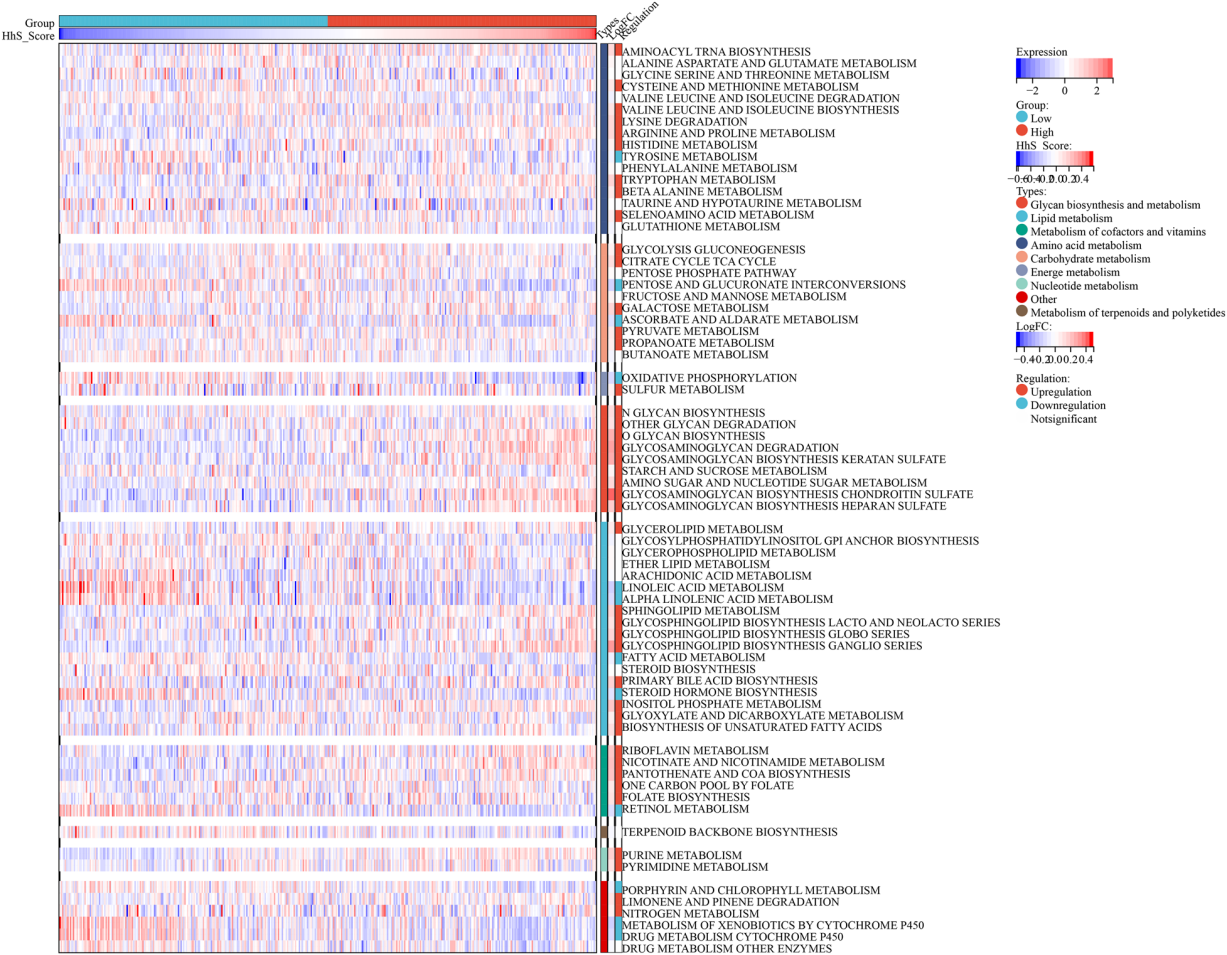
Correlation analysis between HhS and bladder cancer metabolism. Heatmap of metabolism signatures in high and low HhS score group patients.

Next, the association of HhS with metabolic pathways was explored (Fig. 6). The patients in the high HhS group showed extensive upregulation of metabolic activity. Among all 70 KEGG metabolic pathways, 42 were upregulated and 12 were downregulated. Patients with high HhS have significantly activated glycolysis, pentose phosphate pathway, and nucleotide metabolism, which are considered to be the dependent pathways of malignant tumor progression^22^. And the pathways related to drug metabolism, including "Xerobiotics’ metabolism by cytochrome p450" and "Drugs’ metabolism cytochrome p450", were inhibited, which suggests that related drugs may be more effective for patients with high HhS.

### HhS relation to tumor immune microenvironment (TIME)

**Figure 7:**
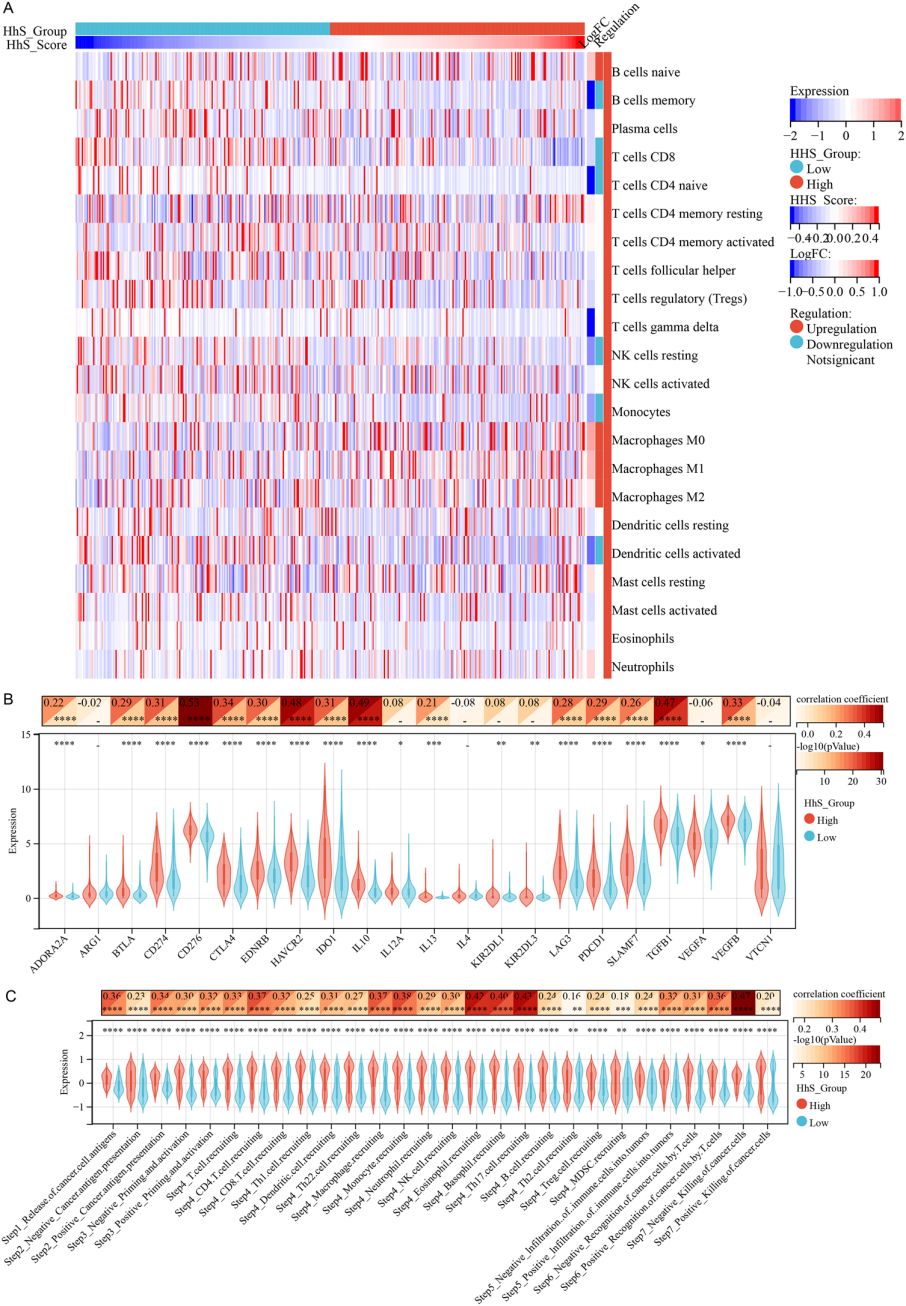
Correlation analysis between UCPs and tumor immune microenvironment. (**A**) Heatmap of immune cells in high and low HhS score group patients. (**B**–**C**) Correlation heatmap (up) of (**B**) immune checkpoints and (**C**) immune cycle score correlation with HhS and violin plot (down) in high and low HhS groups. *P < 0.05, **P < 0.01, ***P < 0.001 and ****P < 0.0001.

We evaluated the expression levels of immune checkpoint genes, the activity of the cancer immunity cycle, and the infiltration status of tumor-infiltrating immune cells to investigate the possible link between HhS and immunological features (Fig. 7). First, the CIBERSORT algorithm was used to deconvolute the infiltration of immune cells in TME. It was ascertained that patients with high expression of HhS had less infiltration of memory B cells, naive CD4 + T cells, CD8 + T cells, monocytes, resting NK cells, and the activated dendritic cells (DCs), and higher infiltration of M0 macrophages, naive B cells, M2 macrophages and M1 macrophages (Fig. 7A). And HhS scores were positively linked to a majority of immune checkpoints (Fig. 7B). Interestingly, the results of immunity cycle analysis deduced that HhS score was positively linked to positive and negative regulation scores of all immune cycle steps (Fig. 7C).

**Figure 8:**
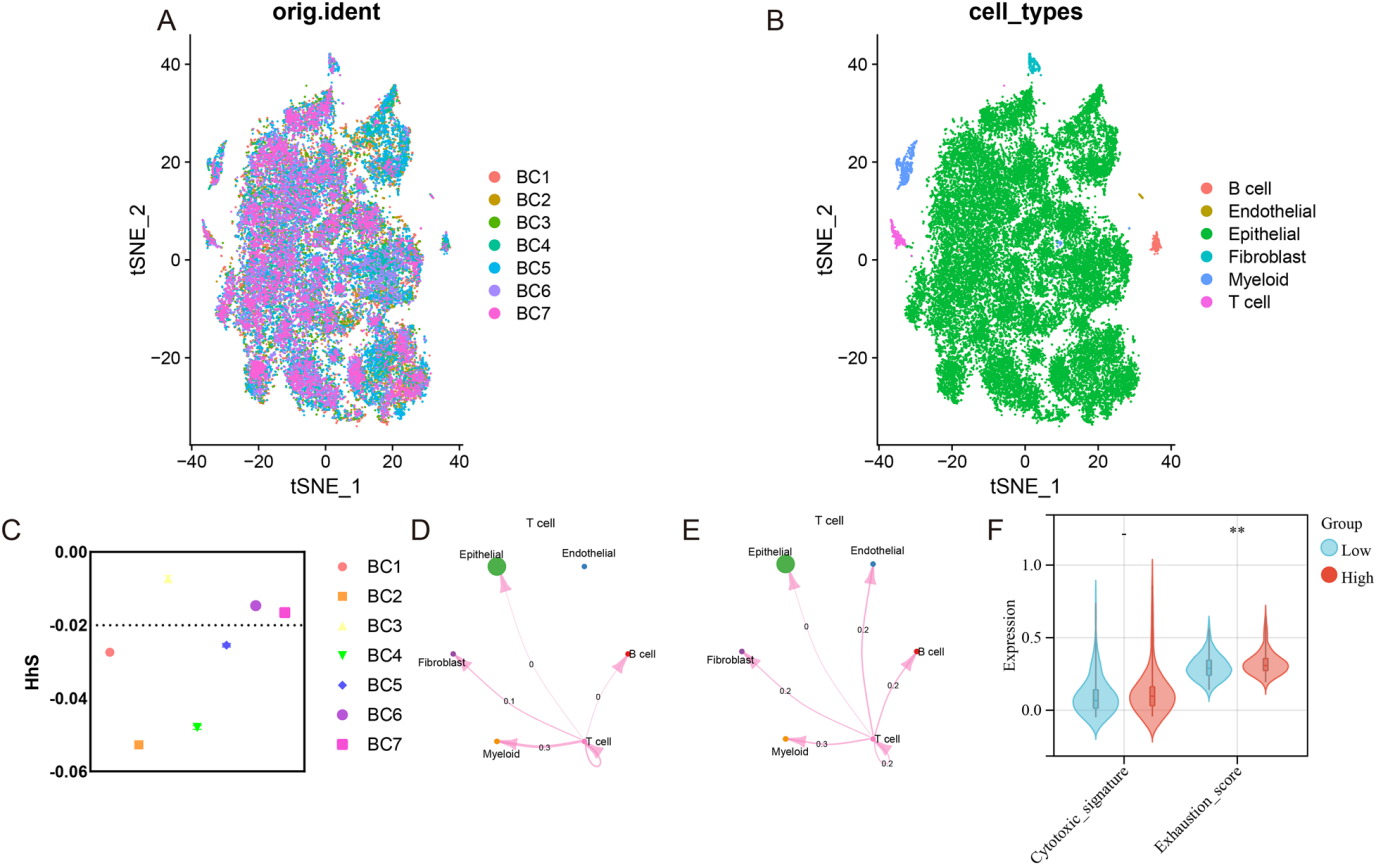
High HhS implies an Immunosuppressed tumor microenvironment in the single cell cohort. (**A**, **B**) t-SNE plot showing the composition of (**A**)7 bladder cancer samples and (**B**) 6 main subtypes. (**C**) Distribution in the HhS of seven bladder cancer samples (divided into 2 patterns). (**D**–**E**) Circos plots displaying putative ligand-receptor interactions between T cells and other cell clusters from (**D**) high-HhS and (**E**) low-HhS group. The brand links pairs of interacting cell types, and corresponding number of events were labeled in the graph. (**F**) Differences in cytotoxic score and exhausted scoreof T cells between high and low HhS groups. *P < 0.05, **P < 0.01, ***P < 0.001 and ****P < 0.0001.

To further explore the function of HhS in TIME, scRNA-seq data of seven bladder cancer patients were obtained from GSE135337 and were annotated (Fig. 8A, B). Seven samples were classified into a low-HhS group and a high-HhS group as per the average HhS expression of samples (Fig. 8C). We used CellChat to infer the presumed intercellular interaction based on ligand-receptor signal transduction and observed that the T cell signal in the high HhS group was weakened (Fig. 8D, E). Consistently, T cells in the high HhS group showed higher exhaustion scores (Fig. 8F).

These results suggest a significant antagonistic effect between immune invasion and immunosuppression in tumor tissues with high HhS and an overall predominance of immunosuppression.

### HhS predicts therapeutic opportunities

**Figure 9:**
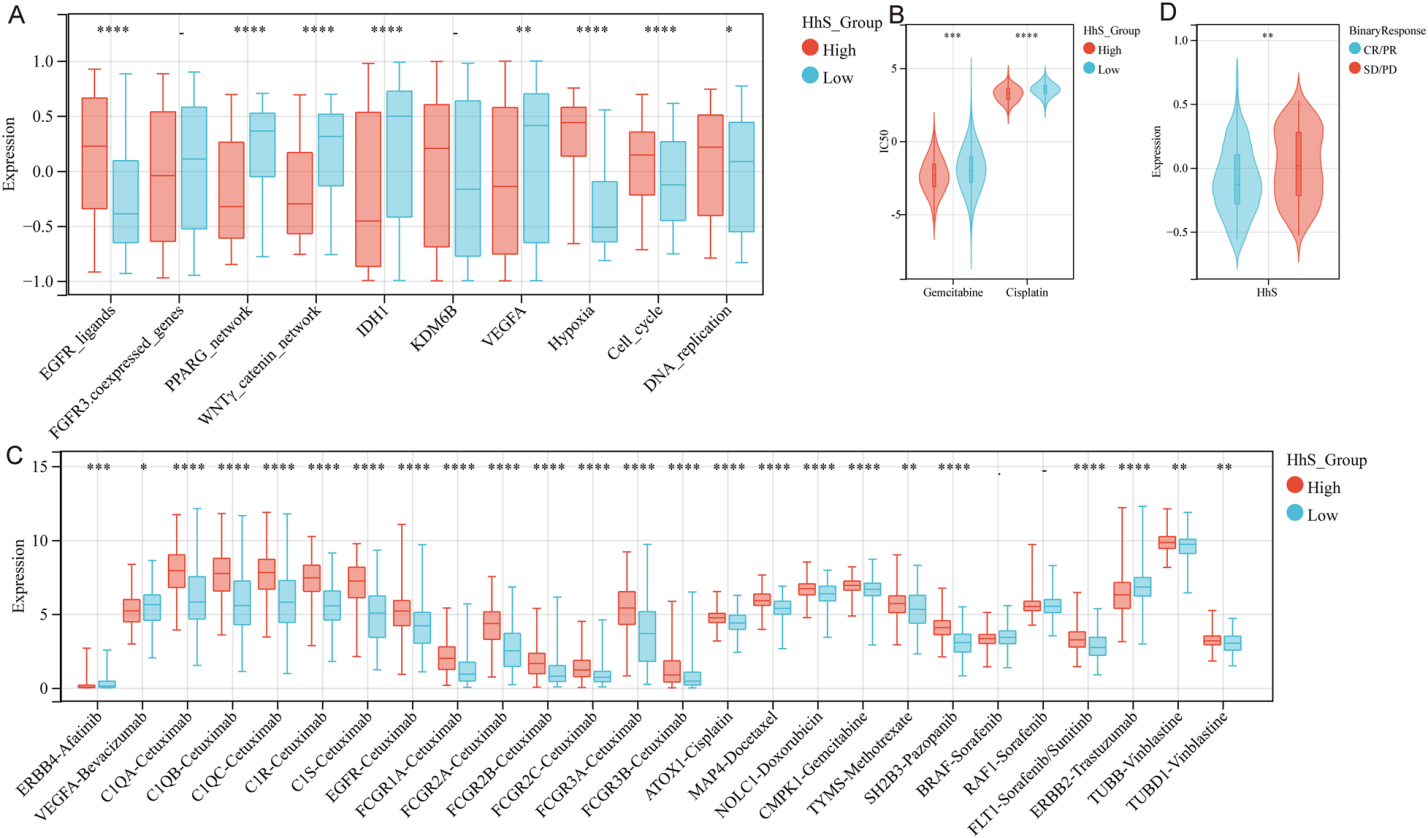
Correlation between HhS and the therapeutic response to several therapies in bladder cancer (**A**–**C**) Differential expression of (**A**) enrichment scores of therapeutic signatures such as targeted therapy and radiotherapy, (**B**) IC50 of gemcitabine and Cisplatin, and (**C**) drug-target genes in high and low HhS groups. (**D**) HhS score in patients with different clinical response of cancer immunotherapy in the IMvigor210 cohort.

Next, the association of HhS with chemotherapy, radiotherapy, and numerous targeted therapies' clinical responses was analyzed. The high HhS group showed higher enrichment scores for EGFR network (EGFR ligands) and radiotherapy-predicted pathways (cell cycle, hypoxia, and DNA replication), while the low HhS group expressed higher enrichment for immune-inhibited oncogenic pathways (PPARG network, WNTγ catenin network, IDH1, VEGFA) (Fig. 9A). The IC50 values calculated by pRRophetic revealed that the high HhS group had higher sensitivity to gemcitabine and cisplatin, chemotherapeutic agents commonly used in bladder cancer (Fig. 9B). In addition, results from the Drugbank database showed that the high HhS group expressed higher levels of EGFR inhibitors as well as chemotherapeutic agents-related targets (Fig. 9C). In addition, data from the IMvigor210 cohort showed that patients who were effective after receiving immunotherapy expressed lower HhS (Fig. 9D).

The mutation profile was analyzed in the TCGA-BLCA cohort (Fig. S2). The low HhS group showed higher overall mutation frequency and had higher mutation frequencies of RYR2, FGFR3, and ELF3.

### HhSRS development and validation

**Figure 10:**
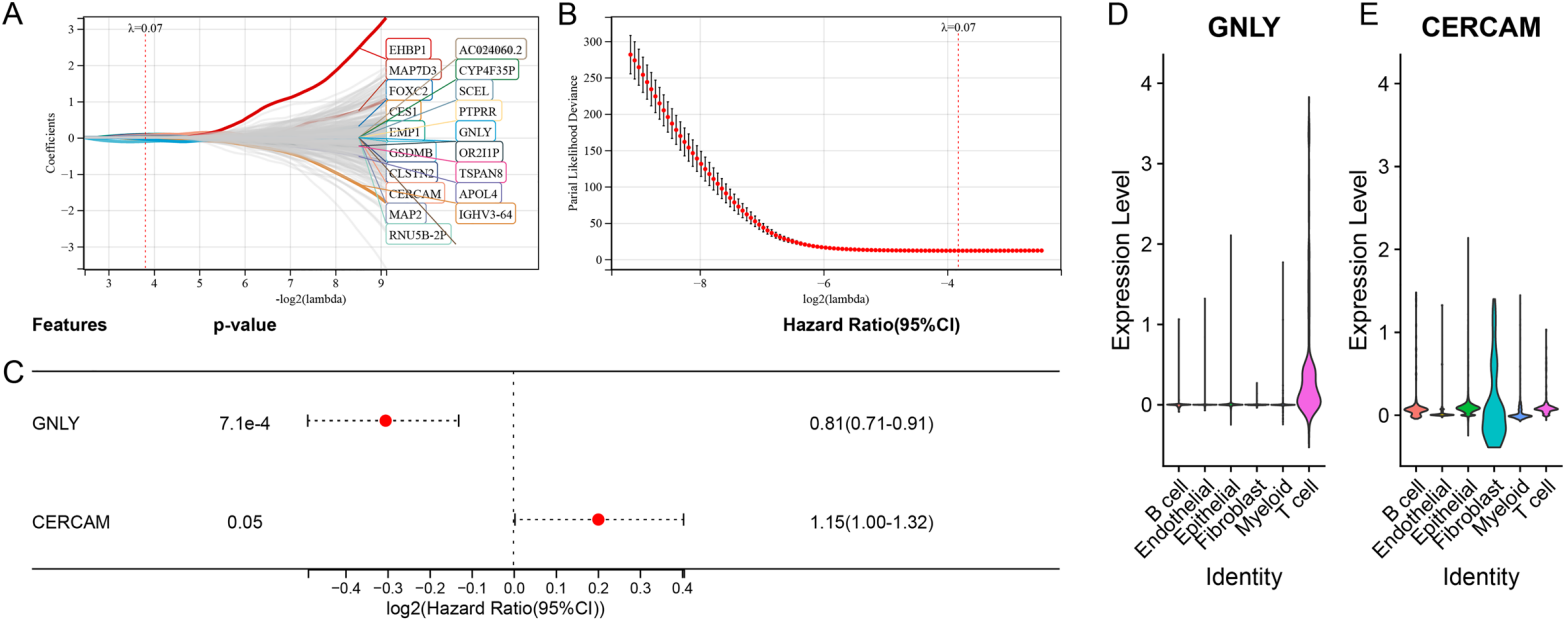
Construction of the HhSRS (**A**, **B**) LASSO regression method was used to screen candidate genes. (**C**) Multivariate Cox regression to screen key genes thoroughly. (**D**–**E**) Expression of (**D**) GNLY and (**E**) CERCAM of different cell types in scRNA-cohort.

High- and low-HhS groups were compared using univariate Cox regression to identify 1290 DEGs from DEGs, and HhSRS was then developed. LASSO regression analysis (Fig. 10A, B) and multivariate Cox regression (Fig. 10C) analysis were used to reduce the overfitting of the HhSRS. Ultimately, 2 genes were recognized to derive the HhSRS: Risk score = 0.14135* cerebral endothelial cell adhesion molecule (CERCAM)-0.21481* granulysin (GNLY). ScRNA-seq analysis showed that GNLY was primarily expressed in T cells (Fig. 10D) and CERCAM was mainly shown in fibroblasts (Fig. 10E).

**Figure 11:**
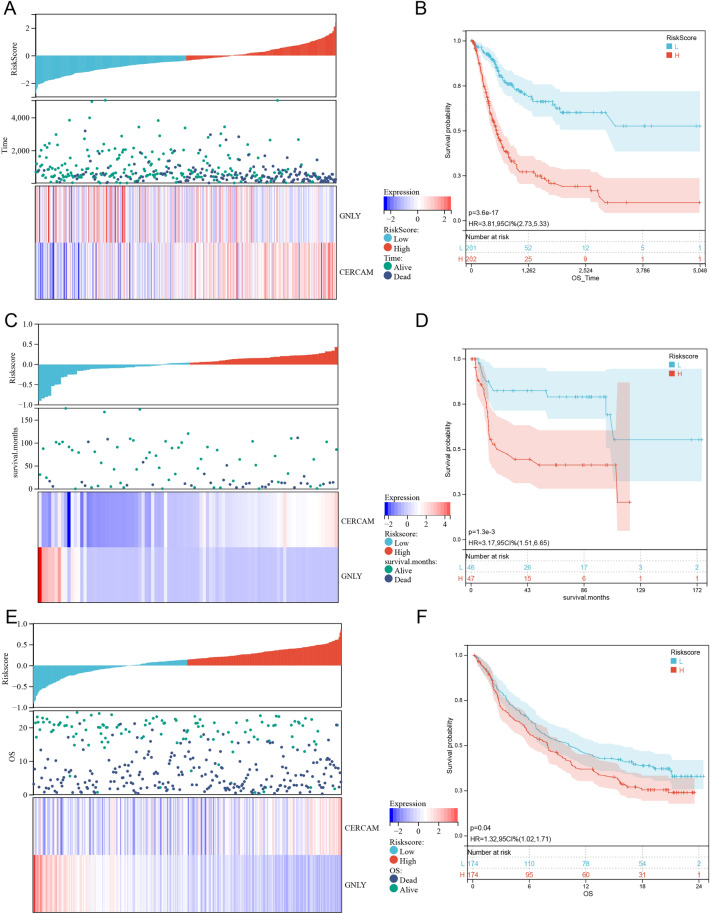
Validation of the HhSRS Risk score analysis and Kaplan–Meier analysis of HhSRS in (**A**, **B**) TCGA cohort, (**C**, **D**) GSE31684 cohort and (**E**, **F**) IMvigor210 cohort.

Patients were divided into two groups based on their HhSRS median (Fig. 11A). Then, the predictive efficacy of HhSRS was confirmed using KM survival curves (Fig. 11B). Patients in the TCGA-BLCA cohort with a high HhSRS risk experienced a significantly lower chance of survival. The HhSRS was next subjected to test in the GSE31684 cohort, where it also showed promising findings (Fig. 11C, D). In the IMvigor210 cohort, we then re-verified the prognostic impact of HhSRS on immunotherapy, and similarly, patients with low HhSRS had a considerably better prognosis than those with high HhSRS (Fig. 11E, F).

**Figure 12:**
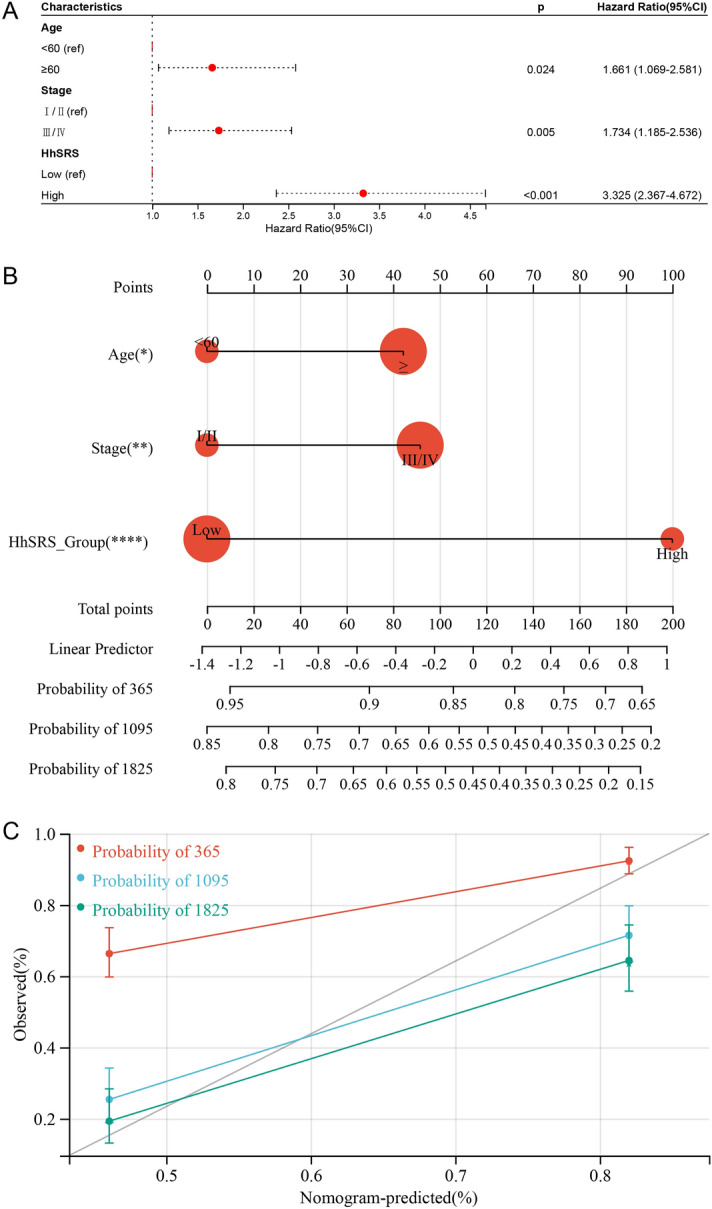
Establishment of the HhSRS related nomogram. (**A**) The forest plots for multivariate regression of clinical factors and HhSRS. (**B**) Nomogram for predicting the OS probability, by setting age, HhSRS group and tumour stage as parameters. (**C**) Calibration curves for verifying the prediction accuracy; red represents the 1-year prediction, blue represents the 3-year prediction, and green represents the 5-year prediction.

We resorted a nomogram to build a predictive model, taking clinicopathological covariates into account, thus establishing a clinically effective technique that could predict the likelihood that a patient would survive. Based on the cox analysis (Fig. 12A), a nomogram to predict OS rates was built (Fig. 12B). Further, the calibration curves showed that the HhSRS-related nomogram accurately predicted the likelihood of survival (Fig. 12C).

## Discussion

Bladder cancer comprises various heterogeneous subtypes, and every subtype possesses its own clinical and biological characteristics^15^. The identification of reliable prognostic biomarkers to pinpoint bladder cancer patients at high risk who might benefit from aggressive treatment represents one of the latest hotspots in the disease^23^. Dysfunction or aberrant activation of the HhS pathway is linked to developmental deformities and cancers^5^. Shin et al. found that when invasive urothelial carcinoma develops, SHH expression is lost inevitably. Inhibiting stromal response to SHH genetically hastens disease progression and shortens the time of survival^24^. As per findings of a few studies, dysregulation of SHH ligand or one of its downstream mediators, for instance, PTCH1, SMO, or GLI1, has been linked to urothelial carcinoma initiation and progression^8,25,26^ and in modulating cancer stem cell activities^27^. Uncontrolled activation of the HhS pathway is linked to tissue healing and tissue homeostasis, whereas regulated activation of the pathway enhances cancer advancement^28^. Numerous small molecules have been identified as HhS pathway antagonists at various levels to date^6^. However, due to technical constraints, most previous studies have been limited to focusing on a few key proteins in HhS rather than the entire pathway.

The fast development of transcriptome, genome, and bioinformatics approaches over the last decade has greatly aided therapeutic options pertaining to cancer via their impact on the discovery of cancer biomarkers and the personalization of cancer therapy^29–31^. The present research confirmed that HhS may be a key factor for bladder cancer-related prognosis and is linked to poor outcomes based on a bioinformatics technique and RNA-seq data. Furthermore, a reliable HhSRS was created for bladder cancer-related prognosis prediction, thus aiding in improving the diagnosis and individualization of treatment for bladder cancer patients.

Molecular subtyping may provide light on the molecular heterogeneity of bladder cancer and can be used to make predictions about patients’ prognoses and responses to therapy^15,21^. The results of this research suggest that Basal bladder cancers have significantly elevated HhS activation. In terms of mechanism, basal bladder cancers have higher invasiveness, which relies on their higher levels of cancer stem cells and EMT characteristics^32^. And sustained activation of HhS contributes to tumor stem cell characteristics and EMT process activation^33,34^. Therefore, blocking the HhS pathway may be a potential approach to improve survival in patients with basal bladder cancer.

Cancer cells have remarkable metabolic plasticity to obtain nutrients for growth and survival under the harshest conditions, with aerobic glycolysis (Warburg effect) being one of the best-known features of tumor metabolic reprogramming^22^. Previous evidence suggests that activation of HhS results in a cellular transition to aerobic glycolysis^35^. Our study shows that patients with high HhS have elevated levels of amino acid metabolism, glycolysis, pentose phosphate pathway, and nucleotide metabolism, which are all fuels required for malignant cell growth and proliferation. Thus, Hh activity regulation is involved in cancer cell metabolic plasticity in migration and metastasis.

Anticancer immunotherapy is vital in the treatment strategy of bladder cancer, and immune checkpoint inhibitors (ICIs), anti-PD1 or anti-PD-L1 antibodies, have exhibited efficacy in clinical trials alone or in conjunction with chemotherapy^36^. Nevertheless, the mechanisms of resistance to these treatments are largely unknown. The association of Hh signaling with immunotherapy has been explored previously. Although ICIs maintained strong anti-tumor activity in the Hh-independent form of medulloblastoma, the work by Pham et al. confirmed that they had low efficacy in the Hh-dependent model^37^. Low immune infiltration and poor response to ICIs in cancer patients are linked to the Wnt pathway^38^, which is mechanistically related to HhS signaling^39^. Consistently, the findings of the presented study affirmed that bladder cancer patients with higher HhS had less CD8 + T cell infiltration and their T cells exhibited a higher depletion profile, in addition, patients who were not sensitive to immunotherapy had Higher HhS scores. Therefore, combination therapy of HhS signaling pathway targeting and immune checkpoint blockers may benefit bladder cancer patients.

Our study identified two genes, namely, GNLY and CERCAM, which were linked to high-risk and low-risk, correspondingly. GNLY, belonging to the saposin-like family of proteins, is a cytolytic and proinflammatory molecule constitutively. GNLY was previously revealed to be also linked to the cytotoxicity and characteristic of effector memory T cells in metastatic melanoma patients^40^. Consistently, the current study also showed that GNLY is specifically expressed in bladder cancer T cells. CERCAM is an adhesion molecule whose expression is at high levels in brain microvasculature, which leukocytes can use to stretch across the blood–brain barrier, regulate leukocyte migration capacity and adhesion, and has been found to be a negative prognostic marker in several tumors^41–43^. Its overexpression promotes cell viability, proliferation, and invasion and is associated with the PI3K/AKT pathway^44^. The presented study found that the expression of CERCAM was higher in fibroblasts than in cancer cells, so further studies are needed to investigate its role in TME.

Our study still had some limitations. Because raw data were gathered from publicly available datasets, the study was retrospective. Further in vitro or in vivo testing is required to confirm the findings. The two independent prognostic genes' fundamental processes were not further investigated. In summary. to confirm the findings and define the putative molecular mechanism at play, functional studies of the two independent prognostic genes were necessary.

## Conclusion

This study demonstrates that HhS is a poor prognostic factor in bladder cancer and mediates the association of bladder cancer with molecular subtypes, metabolic promotion, and immunosuppression. In addition, HhS can predict bladder cancer response to several therapies. Therefore, it seems that targeting HhS might be a useful strategy for treating bladder cancer.

## Supplementary Information

Supplementary Information 1.

Supplementary Information 2.

## Data Availability

The raw data of our study were downloaded from TCGA dataset (http://cancergenome.nih.gov/), GEO dataset (GSE31684, https://www.ncbi.nlm.nih.gov/geo/query/acc.cgi?acc=GSE31684; GSE135337, https://www.ncbi.nlm.nih.gov/geo/query/acc.cgi?acc=GSE135337) and IMvigor210CoreBiologies dataset (http://research-pub.gene.com/IMvigor210CoreBiologies/).

## Abbreviations

Hh: Hedgehog
SHh: Sonic hedgehog
IHh: Indian hedgehog
DHh: Desert hedgehog
Smo: Smoothened
Ptch: Patched
HhS: Hh signaling
TPM: Transcripts per kilobase million
FPKM: Fragments per kilobase of exon per million mapped fragments
MAF: Mutation annotation format
GEO: Gene expression omnibus
scRNA-seq: Single-cell RNA sequencing
MsigDB: Molecular signatures database
KEGG: Kyoto encyclopedia of genes and genomes
DEGs: Differentially expressed genes
GO: Gene ontology
GSEA: Gene set enrichment analysis
FC: Fold change
OS: Overall survival
LASSO: Least absolute shrinkage and selection operator
BP: Biological process
CC: Cellular component
MF: Molecular function
TIME: Tumor immune microenvironment
CERCAM: Cerebral endothelial cell adhesion molecule
GNLY: Granulysin
ICIs: Immune checkpoint inhibitors
