# Supplementary material for: The prognostic value of hedgehog signaling in bladder cancer by integrated bioinformatics
Source: Sci Rep. 2023 Apr 17;13:6241. doi: 10.1038/s41598-023-33140-z (PMC10110581; doi:10.1038/s41598-023-33140-z)
Supplement: Supplementary file 1 — Supplementary Information 1. [file 41598_2023_33140_MOESM1_ESM.docx]

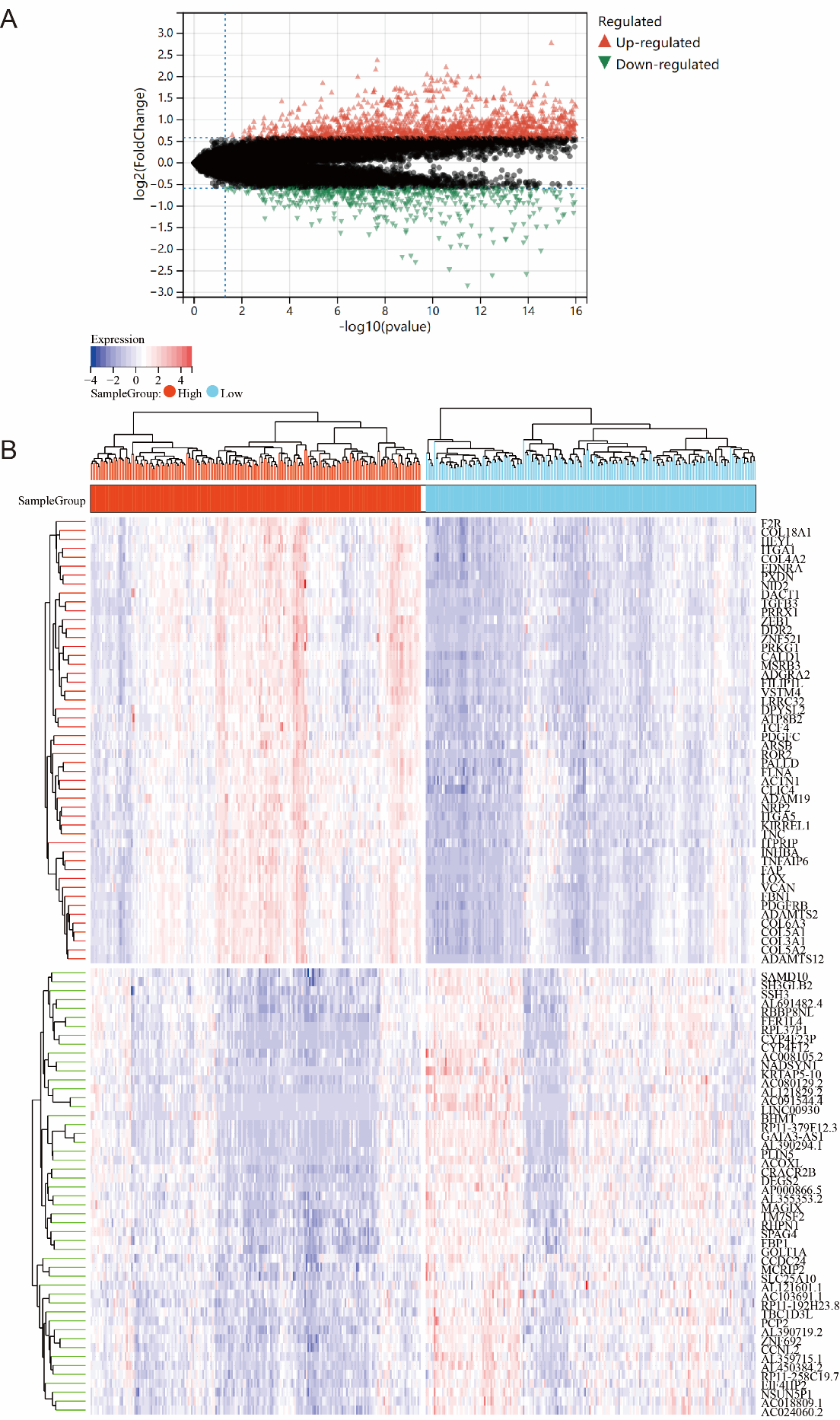


### Figure S1. Identification of DEGs between the HhS groups.

(A) Volcano plot of DEGs. (B) Heatmap of DEGs; the change from red to blue represents a gradual decrease in relative gene expression


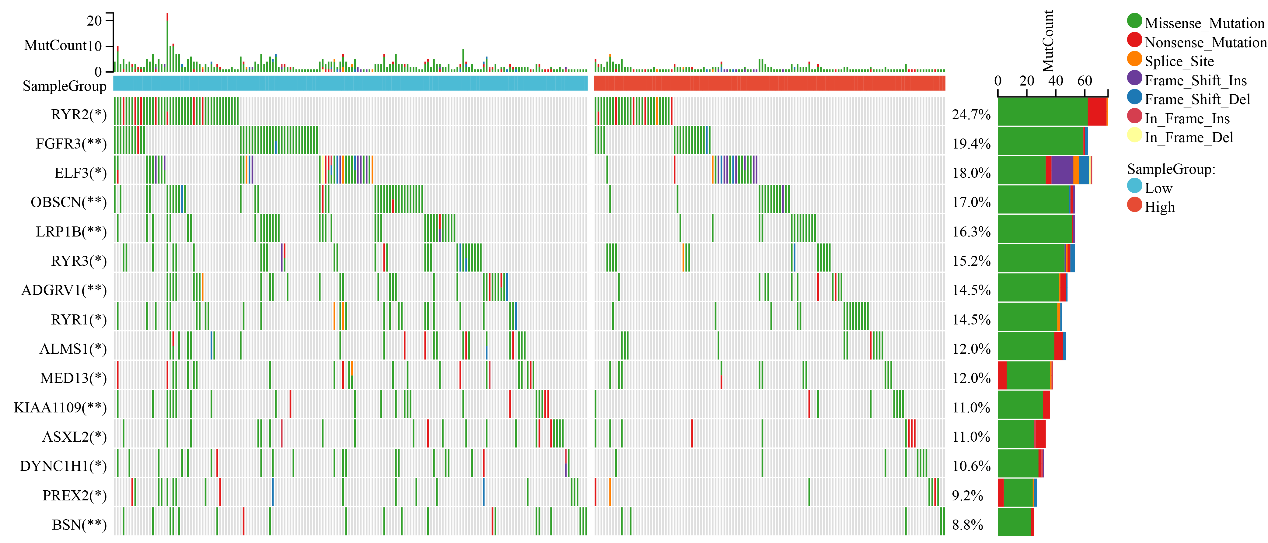


### Figure S2. HhS and mutation landscape.

Comparison of the mutation landscape between groups with high and low HhS score.
